# Lercanidipine Enhances Cisplatin Activity: Dual Anticancer and Anti-Inflammatory Effects via Caspase Activation and MAPK Inhibition

**DOI:** 10.3390/ph18050651

**Published:** 2025-04-29

**Authors:** Tugce Uskur, Sevde Nur Biltekin, Gokhan Faikoglu, Kubra Saygisever-Faikoglu, Barkın Berk

**Affiliations:** 1 Department of Medical Pharmacology, Faculty of Medicine, Kırklareli University, Kırklareli 39100, Türkiye; tugceuskur@gmail.com; 2 Department of Pharmaceutical Microbiology, School of Pharmacy, Istanbul Medipol University, İstanbul 34810, Türkiye; 3 Department of Pharmaceutical Chemistry, Faculty of Pharmacy, Okan University, İstanbul 34959, Türkiye; gokhan.faikoglu@okan.edu.tr (G.F.); barkin.berk@okan.edu.tr (B.B.); 4 Department of Medical Pharmacology, Faculty of Medicine, Cerrahpasa-Istanbul University, İstanbul 34098, Türkiye; ksaygisever@hotmail.com

**Keywords:** lercanidipine, cisplatin, cell culture, calcium channels, cancer

## Abstract

**Background/Objectives**: Lercanidipine is a third-generation dihydropyridine calcium channel blocker. In addition to their well-established cardiovascular effects, calcium channel blockers are increasingly recognized for their therapeutic potential in various cancers. This study aimed to investigate the potential anticancer effects of lercanidipine on cancer cell lines—particularly in combination with cisplatin—by assessing parameters such as cell viability (MTT assay), proliferation, MAPK pathway activity, caspase enzyme levels, and TNF-α expression. **Methods**: In this study, the effects of lercanidipine, both alone and in combination with cisplatin, on cell viability were evaluated using the MTT assay in MCF-7, SH-SY5Y, PC3, and HEK293 cell lines. To assess intracellular signaling and apoptotic pathways, MAPK inhibition, as well as caspase-3 and caspase-8 activities, were measured using ELISA. Additionally, to evaluate the anti-inflammatory potential, TNF-α levels in LPS-stimulated RAW264.7 cells were analyzed via. **Results**: The study revealed that lercanidipine showed significant cytotoxic effects, particularly in SH-SY5Y and PC3 cancer cell lines, while it did not induce a 50% loss of viability in healthy HEK293 cells. When combined with cisplatin, lercanidipine enhanced cytotoxicity by 2.7-fold in neuroblastoma (SH-SY5Y) cells, 1.6-fold in breast cancer (MCF7) cells, and 1.9-fold in prostate cancer (PC3) cells. MAPK activity was inhibited by 83.6% at 20 μM lercanidipine, while dose-dependent increases in caspase-3 and caspase-8 activities were observed. Additionally, lercanidipine decreased TNF-α levels in LPS-stimulated RAW264.7 cells, indicating its potential anti-inflammatory effect. **Conclusions**: In conclusion, lercanidipine demonstrated selective anticancer effects in cancer cell lines and showed synergistic cytotoxicity when combined with cisplatin. It also significantly inhibited MAPK signaling, activated apoptotic caspases, and reduced TNF-α levels, suggesting potential anti-inflammatory activity. These findings highlight lercanidipine’s potential for repurposing as an adjunct in cancer therapy.

## 1. Introduction

Lercanidipine (LRD) is a third-generation calcium channel blocker with a 1,4-dihydropyridine (DHP) structure. Similar to other drugs in this class, it reversibly blocks voltage-dependent Ca^2+^ influx through L-type channels in the cell membrane, leading to peripheral vasodilation and, consequently, a reduction in blood pressure [1]. In addition to their cardiovascular effects, DHPs are known to possess various pharmacological effects, including anticonvulsant, antimycobacterial, antioxidant, antidiabetic, antituberculosis, and anti-inflammatory properties [2,3,4]. According to preclinical studies, LRD has demonstrated antiatherogenic potential independent of its blood pressure-lowering effects and may protect against end-organ damage [5]. In addition, it has shown renoprotective properties in patients with diabetes and renal failure [6]. More recent findings suggest that LRD also exhibits anti-inflammatory, antioxidant, and antiapoptotic properties beyond its calcium channel blockade [7,8]. Calcium homeostasis plays a critical role in many diseases and biological mechanisms, in addition to blood pressure regulation [9]. Its association with cancer, in particular, has highlighted the potential anticancer effects of the DHP class of drugs. Disruptions in calcium homeostasis play a key role in cancer biology. Lercanidipine, a third-generation calcium channel blocker, has demonstrated anti-inflammatory and cytoprotective properties, making it a potential candidate for combination therapy with chemotherapeutic agents. Recent findings suggest that calcium channel blockers may enhance the efficacy of traditional chemotherapy agents by modulating apoptosis and reducing drug resistance mechanisms [10].

Intracellular calcium ions (Ca^2+^) serve as crucial second messengers in regulating cell proliferation, gene transcription, cell migration, and cell death. Mounting evidence indicates that intracellular Ca^2+^ homeostasis is disrupted in cancer cells, and this disruption plays a critical role in tumor initiation, angiogenesis, progression, and metastasis. Dysregulation of Ca^2+^ channels, transporters, and pumps may act as a key driver in carcinogenesis. Studies in this field further suggest that targeting Ca^2+^ signaling pathways could contribute to the development of novel chemotherapeutic agents for various types of cancer [11,12]. Numerous preclinical studies and gene expression data have demonstrated that calcium channels are highly expressed in various types of cancer and play a crucial role in regulating cellular Ca^2+^ homeostasis [13,14].

In addition, several compounds in the 1,4-dihydropyridine (DHP) class have demonstrated anticancer effects. One of the most important pharmacological potentials of DHPs was first identified in a 1980 study, which reported their ability to reverse multidrug resistance (MDR) in cancer cell lines. Furthermore, several dihydropyridine compounds with modified chemical structures have been found to enhance drug efficacy in doxorubicin-resistant cancer cells by inhibiting P-glycoprotein (P-gp) function while also exhibiting direct anticancer effects [15,16].

Recent studies have suggested that the effects of lercanidipine, beyond calcium channel blockade, may play a significant role in cancer cells. Its antiapoptotic and antioxidant properties have been reported to reduce cell proliferation across various cancer types and target the survival mechanisms of cancer cells. Moreover, an increasing number of studies indicate that combining cisplatin with calcium channel blockers can yield synergistic effects. For instance, the combined use of verapamil and cisplatin has exhibited anti-metastatic effects in chemoresistant cancer cells [17]. Additionally, calcium channel blockers such as manidipine have been shown to enhance chemosensitivity, suppress cell proliferation, and reduce stem cell properties in ovarian cancer stem cells when administered in combination with cisplatin [18]. These findings suggest that lercanidipine may likewise exhibit a synergistic effect when used alongside cisplatin. Furthermore, growing evidence points to lercanidipine’s ability to inhibit cell proliferation by suppressing the MAPK signaling pathway [19]. Overall, these data indicate that lercanidipine not only provides cardiovascular protection but also holds potential as a therapeutic agent in cancer treatment.

In this study, we aimed to investigate the potential anticancer effects of lercanidipine on cancer cell lines, particularly in combination with cisplatin, and to examine its possible impact on parameters such as cell viability (MTT), proliferation, MAPK pathway activity, caspase enzyme levels, and TNF-α.

## 2. Results

### 2.1. Cytotoxic Assay

In this study, the cytotoxic effects of lercanidipine and cisplatin were evaluated using the MTT assay. The cytotoxic effects of agents were investigated on both the healthy cell line HEK293 and the cancer cell lines SH-SY5Y, PC3, and MCF7. Lercanidipine was applied to the cells at varying concentrations (1, 5, 10, 20, 50, 100, and 150 μM), and their effects were assessed after 48 h of incubation. The concentrations that inhibited 50% of cell viability, referred to as the half-maximal inhibitory concentration (IC_50_), were determined and are presented in Table 1.

The results revealed that lercanidipine exhibited a particularly high cytotoxic effect on brain tumor cells. The IC_50_ value of lercanidipine on the SH-SY5Y cell line was calculated as 31.48 μM. Additionally, lercanidipine demonstrated a pronounced cytotoxic effect on prostate cancer cells, with an IC_50_ value of 88.60 μM. Although lercanidipine was not as effective as cisplatin, which served as the positive control, in terms of cytotoxicity on cancer cells, its selective toxicity is a significant finding. Cisplatin exhibited high toxicity toward healthy HEK293 cells, with an IC_50_ value of 2.98 μM. In contrast, lercanidipine did not reduce HEK293 cell viability by 50%, even at its maximum concentration (150 μM).

To investigate the effects of combining lercanidipine with cisplatin, cytotoxicity assays were conducted on both cancerous and healthy cell lines. The results of combined treatments were compared to those of individual applications. Notably, the combination of lercanidipine and cisplatin demonstrated a 2.7-fold increase in cytotoxic effect on SH-SY5Y neuroblastoma cells compared to lercanidipine alone. Similarly, the combined application of lercanidipine and cisplatin showed a 1.6-fold increase in cytotoxicity on MCF7 breast cancer cells and a 1.9-fold increase in PC3 prostate cancer cells compared to individual treatments (Table 2).

### 2.2. MAPK Enzyme Activity

The MAPK enzyme inhibition of the lercanidipine was evaluated using a commercially purchased kit. In this context, the % MAPK inhibition value of lercanidipine at a concentration of 20 μM was found to be 83.60. Its effect, comparable to the positive control provided with the commercial kit, is considered a highly significant finding. The results are shown in Table 3.

### 2.3. Caspase Activity Assay

**Figure 1:**
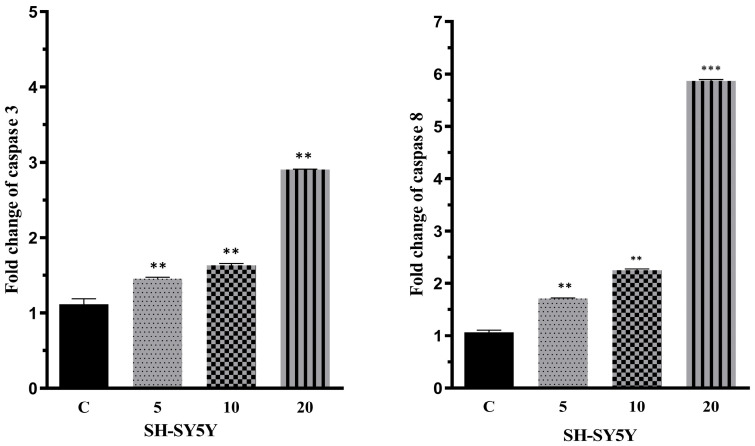
Effects of lercanidipine on caspase-3 and caspase-8. All data are statistically significant (mean ± SD, n = 3, ** *p* < 0.01, *** *p* < 0.001, vertical bars indicate standard deviation values).

The effects of lercanidipine on caspase-3 and caspase-8 proteins, which are involved in different pathways of apoptosis mechanisms, were investigated. SH-SY5Y cells were treated with lercanidipine at concentrations of 5 μM, 10 μM, and 20 μM, followed by cell lysis. The effects of the compound on caspase-3 and caspase-8 enzyme activities were determined using commercial ELISA kits. Untreated cells were used as the control group. The obtained results indicated that all data were statistically significant (at least * *p* < 0.05). The levels of caspase-3 and caspase-8 proteins in SH-SY5Y cells treated with lercanidipine and the positive control are presented in Figure 1.

According to the results, lercanidipine was found to induce caspase-3 and caspase-8 activation in a concentration-dependent manner. At concentrations of 5 μM, 10 μM, and 20 μM, lercanidipine increased caspase-3 activity approximately 1.4-fold, 1.6-fold, and 2.9-fold, respectively, compared to the control group. Similarly, lercanidipine increased caspase-8 activity approximately 1.7-fold, 2.2-fold, and 5.8-fold at concentrations of 5 μM, 10 μM, and 20 μM, respectively, compared to the control group.

### 2.4. Results of Anti-Inflammatory Activity

**Figure 2:**
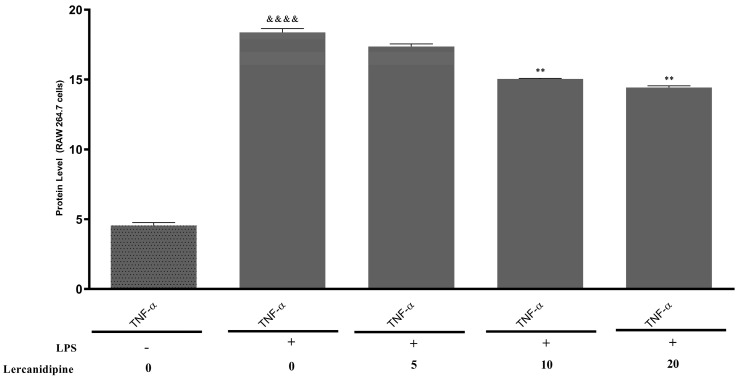
Effects of lercanidipine on TNF-α levels in RAW 264.7 cells with or without LPS stimulation. “^&&&&^” (*p* < 0.0001) indicates significance compared to the non-LPS group. “**” (*p* < 0.01) indicates significance compared to the LPS-treated positive control group.

For the analysis of anti-inflammatory effects, lercanidipine was applied to RAW264.7 cells, and the TNF-α levels in the cells were measured using ELISA kits. Initially, the TNF-α levels were determined in cells that were untreated and in those stimulated with LPS. It was observed that TNF-α levels were significantly induced in LPS-stimulated cells. The difference between the untreated control group and the LPS-stimulated group was substantial, and the results were statistically significant (*p* < 0.0001). When the LPS-stimulated group was considered the positive control, and the effects of lercanidipine at different concentrations (5, 10, and 20 μM) were compared, a concentration-dependent reduction in TNF-α levels was observed. All results are presented in Figure 2.

The findings demonstrated that lercanidipine exhibited a concentration-dependent effect on TNF-α levels. The lowest dose of lercanidipine (5 μM) did not result in a statistically significant reduction in TNF-α levels. However, as the concentration increased, the effect of lercanidipine became more pronounced. At higher concentrations (10 μM and 20 μM), a statistically significant decrease in TNF-α levels was observed compared to the positive control group (LPS-stimulated cells).

## 3. Discussion

Since the 1960s, calcium channel blockers have been used in the treatment of hypertension, and various pharmacological effects of lercanidipine, a member of this drug class, have been identified over time. Lercanidipine, a third-generation dihydropyridine calcium channel blocker, exhibits not only antihypertensive effects but also neuroprotective and vasodilatory effects [20], antiatherogenic properties [5], renoprotective properties [6], and anti-inflammatory, antioxidant, and antiapoptotic effects [7,8,21]. The relationship between calcium homeostasis, calcium channel blockers, and cancer highlights the importance of investigating the potential anticancer effects of lercanidipine [13,14,19].

MTT assay results revealed that lercanidipine exerted a significant cytotoxic effect on brain tumor cells (IC_50_ value is 31.48). Additionally, it exhibited cytotoxic activity against prostate cancer cells, with a more pronounced effect compared to that observed in breast cancer cells. Nonetheless, in both cell lines, the cytotoxic potency of lercanidipine remained inferior to that of cisplatin. Additionally, it was observed that lercanidipine did not reduce cell viability by 50% in healthy HEK293 cells. This selective cytotoxicity suggests that lercanidipine may have potential as a therapeutic agent for the treatment of brain tumors. To examine the effects of combining lercanidipine with cisplatin, a widely used chemotherapeutic agent, cytotoxicity assays were conducted, and the combination of lercanidipine and cisplatin demonstrated an increase in cytotoxic effect on SH-SY5Y neuroblastoma cells, MCF7 breast cancer cells, and PC3 prostate cancer cells.

The observed enhancement in cisplatin’s cytotoxic effects when combined with lercanidipine provides promising insights into potential combination therapies for cancer treatment. These results contribute significantly to the literature, offering a new perspective on the adjunctive use of calcium channel blockers in oncology. Previous studies have demonstrated that calcium channel blockers, such as verapamil and manidipine, can enhance the efficacy of chemotherapeutic agents by modulating drug resistance and apoptosis pathways. The synergistic effects observed in this study further support the hypothesis that lercanidipine may have a similar role in cancer therapy. However, compared to other calcium channel blockers, there is currently limited data on lercanidipine’s anticancer mechanisms, necessitating further comparative studies.

Further research is warranted to elucidate the underlying mechanisms and explore clinical applications. While these findings highlight the potential of lercanidipine in oncology, further studies are needed to confirm its effects in preclinical models and clinical settings. Future investigations should focus on evaluating its pharmacokinetics, bioavailability, and potential interactions with standard chemotherapeutics in vivo.

Lercanidipine, a dihydropyridine calcium channel blocker primarily used for hypertension management, has demonstrated potential anticancer properties in recent studies. Research indicates that lercanidipine can enhance the cytotoxic effects of proteasome inhibitors like bortezomib in various solid tumor cell lines by inducing paraptosis, a form of programmed cell death characterized by extensive vacuolation derived from the endoplasmic reticulum and mitochondria [22]. This synergistic effect is associated with increased endoplasmic reticulum stress and mitochondrial calcium overload, leading to cancer cell death. Additionally, lercanidipine has been observed to alleviate doxorubicin-induced lung injury by modulating oxidative stress and apoptotic pathways, suggesting its protective role against chemotherapy-induced toxicity [23]. These findings highlight lercanidipine’s potential as an adjunctive agent in cancer therapy, enhancing the efficacy of existing treatments and mitigating adverse effects. However, further clinical studies are necessary to fully elucidate its anticancer mechanisms and therapeutic applications.

Lercanidipine is a dihydropyridine calcium channel blocker primarily used for hypertension management. Currently, there is no substantial evidence in the scientific literature to suggest that lercanidipine enhances the efficacy of anticancer drugs. However, some studies have explored the potential of other calcium channel blockers in cancer therapy. For instance, a study by Tyagi et al. [24] investigated the synergistic anticancer effects of silibinin, a natural compound, with conventional cytotoxic agents such as doxorubicin, cisplatin, and carboplatin against human breast carcinoma cells. The study found that silibinin enhanced the therapeutic potential of these chemotherapeutic drugs in both estrogen-dependent and -independent human breast carcinoma cells [24]. While these studies provide insights into the interactions between certain compounds and anticancer drugs, they do not specifically address lercanidipine. Therefore, more research is needed to determine any potential role of lercanidipine in cancer treatment.

In our in vitro experiments, the concentrations of lercanidipine used (5–20 µM) are higher than the therapeutic plasma concentrations observed clinically, which are generally in the nanomolar range. However, such concentrations are commonly used in cell culture studies to compensate for factors such as extensive plasma protein binding and limited intracellular drug availability. Lercanidipine is a highly lipophilic molecule with over 98% protein binding, which significantly reduces its free plasma concentration in vivo [25]. Therefore, higher concentrations may be required to observe pharmacological effects in vitro. Similar approaches have been employed in previous studies investigating other calcium channel blockers in cancer models [22].

Kinase enzymes, particularly those within the MAPK (Mitogen-Activated Protein Kinase) signaling pathway, play critical roles in cellular processes such as proliferation, differentiation, and stress response. Dysregulation of MAPK activity is implicated in numerous pathological conditions, including cancer, making this pathway a promising therapeutic target. Preliminary findings from experimental evaluations suggest that lercanidipine, at a concentration of 20 μM, inhibits MAPK activity by 83.60%, as assessed using a commercially available enzyme inhibition assay. Notably, the 20 µM concentration was selected for our mechanistic evaluations because preliminary cytotoxicity assays indicated that higher lercanidipine doses (50 µM and above) led to excessive cell death. Using 20 µM as the maximal concentration ensured that the observed effects on MAPK activity and apoptosis were not confounded by non-specific cytotoxicity, thereby allowing a clearer interpretation of lercanidipine’s molecular effects. This level of inhibition, comparable to the positive control included in the assay, highlights the potential pharmacological significance of this observation. Although no existing studies in the current literature explicitly investigate the MAPK inhibitory effects of lercanidipine, its structural similarity to other dihydropyridine derivatives raises the possibility of interactions with intracellular signaling pathways. Further investigations, particularly those employing in vitro kinase activity assays, in vivo models, and molecular docking studies, are essential to validate this observation and elucidate the underlying mechanisms of MAPK inhibition. This finding not only contributes to a deeper understanding of lercanidipine’s pharmacological profile but also raises the possibility of repurposing calcium channel blockers in the context of diseases driven by MAPK pathway dysregulation. Future research efforts should aim to confirm these results in broader experimental settings and assess the clinical implications of lercanidipine’s off-target effects.

Caspases play a critical role in apoptosis mechanisms, and the induction of caspase-3 and caspase-8 has been associated with cancer cell death and improved therapeutic outcomes [26,27]. In this study, a caspase activity assay was conducted to investigate the effects of lercanidipine on caspase-3 and caspase-8 proteins, and it was observed that caspase-3 and caspase-8 were induced in parallel with the applied concentration of lercanidipine. The data obtained from our caspase activity assays suggest that lercanidipine may act as a potential anticancer agent by inducing apoptosis in cancer cells.

As part of our study, the anti-inflammatory effects of lercanidipine were investigated, and a statistically significant reduction in TNF-α levels was observed at high doses. For the first time in 2006, it was demonstrated that lercanidipine could reduce low-grade systemic inflammation in patients with essential hypertension [28]. Recognizing this potential effect of lercanidipine, researchers have demonstrated its anti-inflammatory properties using various methods [8,29,30]. Since 1863, an increasing number of studies have investigated the relationship between inflammation and cancer [31]. Moreover, the significant impact of inflammatory cells on tumor development is well established [32]. In this context, in addition to its effects, lercanidipine’s anti-inflammatory properties make it a promising candidate for cancer treatment.

It is well established that chronic inflammation plays a pivotal role in tumor development and progression [31,32]. Pro-inflammatory mediators in the tumor microenvironment, such as TNF-α, can promote cancer cell proliferation, angiogenesis, and metastasis, thereby facilitating tumor growth. Consequently, anti-inflammatory interventions are considered a valuable strategy in cancer therapy to disrupt this inflammation–cancer axis. In our study, the observed decrease in TNF-α levels with lercanidipine treatment underscores a key mechanism by which lercanidipine may exert anticancer effects. By attenuating an important inflammatory cytokine, lercanidipine could potentially render the tumor microenvironment less supportive of cancer progression. This dual action—directly inducing cancer cell death while simultaneously reducing pro-tumor inflammation—highlights the significance of our findings and suggests that lercanidipine’s anti-inflammatory properties complement its direct cytotoxic effects against cancer cells.

## 4. Materials and Methods

### 4.1. Cell Culture Conditions

In this study, the cytotoxic effects of lercanidipine were evaluated against several cell lines: MCF-7 (human breast cancer, ATCC^®^ HTB-22™), SHSY5Y (human neuroblastoma cancer, ATCC^®^ CRL-2266), PC3 (human prostate cancer, ATCC^®^ CRL-1435), and HEK293 (human embryonic kidney, ATCC^®^ CRL-1573). All mediums were supplemented with 10% (*v*/*v*) fetal bovine serum (FBS), 1% (*v*/*v*) antibiotic–antimycotic solution (containing 100 U/mL penicillin, 100 μg/mL streptomycin, and 0.25 μg/mL amphotericin B), and 1% (*v*/*v*) non-essential amino acids. The cultures were maintained at 37 °C in a humidified atmosphere containing 5% CO_2_, with cell passages performed every 3 days [33].

### 4.2. MTT Assay

The cytotoxic effects of lercanidipine within the scope of this study were evaluated using healthy human cell line HEK293 as well as several cancer human cell lines (SHSY5Y, PC3, MCF7). The assessment was conducted using the MTT assay, a colorimetric method developed by Mosmann [34].

For this purpose, cells were seeded at a concentration of 1 × 10^5^ cells/mL into 96-well cell culture plates and incubated for 24 h at 37 °C with 90% relative humidity and 5% CO_2_. Following the incubation period, the medium was removed, and the compounds to be tested for toxicity were applied to the cells at varying concentrations (1, 5, 10, 20, 50, 100, 150 μM). The culture plates were then incubated again for 24 h.

At the end of the incubation periods, the medium was removed, and 30 μL of MTT stock solution (5 mg/mL in D-PBS) was added to each well. The plates were incubated for an additional 4 h. To dissolve the formed formazan crystals, 150 μL of DMSO was added to each well, and the plates were agitated at room temperature until the solution was homogeneous. The absorbance of the resulting-colored product was measured at 540 nm using a microplate reader (SpectraMax i3, Molecular Devices, San Jose, CA, USA). The IC_50_ values for lercanidipine and cisplatin were determined using a sigmoidal non-linear regression model based on dose–response curves fitted with GraphPad Prism software. Cell viability was calculated as a percentage relative to the control using the following equation [35] (Equation (1)):

% Viability = (A_s_/A_c_) × 100 (1)

A_s_: Absorbation of sample;

A_c_: Absorbation of control.

The experiments were conducted in triplicate at each time point.

The synergistic effects of lercanidipine and cisplatin on cell proliferation were investigated. SH-SY5Y, PC3, and MCF7 cell lines were treated with lercanidipine and cisplatin at their IC_50_ doses in combination. Following the treatment, cell viability was assessed using the MTT assay described above. To evaluate the synergistic effect, a comparative analysis was conducted by administering the drugs both individually and in combination.

### 4.3. p38MAPK Inhibition Assay

The effects of lercanidipine on different subunits of the p38MAPK enzyme at various time points were investigated as part of this study. The analysis was conducted using a “p38MAPK ELISA Kit”, following the manufacturer’s instructions [33]. Initially, standards, controls, and lercanidipine at 20 µM concentration were added to “striped” microplates and incubated at room temperature for 2 h. Subsequently, p38MAPK (α, β) detection antibodies were added to the wells, and the plates were incubated at room temperature for different durations (30 min, 60 min, 90 min). Following this, “Anti-Rabbit IgG HRP Solution” and “Stabilized Chromogen” were added to develop a blue color. The reaction was terminated by adding a stop solution, and the color change from blue to yellow in the wells was measured at 450 nm using a microplate reader (SpectraMax i3). The experiments were performed in triplicate.

### 4.4. Caspase 3 and Caspase 8 Enzyme Assay

Caspase-3, a protein responsible for caspase stimulation, is one of the effector caspases playing a pivotal role in apoptosis. It is activated through the enzymatic cascade of caspase activation [36]. Caspase-8, on the other hand, is recognized as a key activator of the apoptotic mechanism [37]. Studies have demonstrated a correlation between caspase-8 activation and the stimulation of caspase-3 [38]. Based on this relationship, the effects of lercanidipine on the enzymatic activities of both caspase-3 and caspase-8 in cells were investigated. The effects of lercanidipine on apoptotic protein levels were evaluated using the ELISA method. Experiments were performed following the manufacturer’s instructions using a commercial ELISA kit.

Cells treated with lercanidipine (5, 10 μM) were collected and centrifuged, followed by washing with PBS (pH 7.4). Subsequently, the cells were homogenized in 1X lysis buffer (50 mM HEPES, pH 7.4, 5 mM CHAPS, 5 mM DTT) and disrupted using a sonicator. The homogenate was centrifuged at 16,000× *g* for 20 min at 4 °C, and the supernatants were collected. Samples and standards were placed in ELISA plate wells according to the protocol. The reaction was initiated by adding caspase substrate to each well. The microplate was incubated at 37 °C for 90 min, after which absorbance values were measured at 405 nm using a spectrophotometer. All experiments were conducted in triplicate [39].

### 4.5. Determination of TNF-α Levels

Cytokines are commonly utilized to assess anti-inflammatory activity, with TNF-α being one of the most frequently evaluated pro-inflammatory cytokines (Liu et al., 2017) [40]. In this analysis, TNF-α levels were measured after the application of lercanidipine to RAW264.7 cells using ELISA kits (Elabscience, Houston, TX, USA, and EIAab, Wuhan, China) in accordance with the manufacturer’s instructions. RAW264.7 cells were seeded into 96-well microplates at a density of 1 × 10^5^ cells/mL and incubated for 24 h at 37 °C in a humidified incubator containing 5% CO_2_. TNF-α production in the cells was induced using lipopolysaccharide (LPS). Following induction, lercanidipine at varying concentrations (5, 10, 20 μM) was applied to the cells for 24 h.

To remove dead cells and cellular debris, supernatants were collected after a brief centrifugation at 2000× *g* and subsequently evaluated via the ELISA method. Samples were added to the microplate wells and incubated for 90 min at 37 °C in a 5% CO_2_ incubator. Afterward, 100 μL of “Biotinylated Detection Antibody Working Solution” was added to each well and incubated for 1 h at 37 °C. Following washing steps, 100 μL of “HRP Conjugate Working Solution” was added to each well and incubated for 30 min at 37 °C. The washing steps were repeated, and 90 μL of “Substrate Reagent” was added, followed by a 15 min incubation at 37 °C. The reaction was stopped by adding 50 μL of “Stop Solution” to each well, and absorbance measurements were recorded at 450 nm using a spectrophotometer. The analyses were conducted based on a standard curve. All experiments were performed on three separate occasions, with a minimum of three replicates per condition.

### 4.6. Statistical Analyses

All experiments were performed in three independent replicates, and the data were expressed as mean ± standard deviation (SD). Statistical analyses were performed using ANOVA and post hoc Tukey test with GraphPad Prism software Version 7.02 (La Jolla, CA, USA). A *p*-value of <0.05 was considered statistically significant.

## 5. Conclusions

In conclusion, our study demonstrates that lercanidipine—a 1,4-dihydropyridine calcium channel blocker—exhibits notable anticancer and anti-inflammatory effects in addition to its established antihypertensive action. Lercanidipine selectively reduced the viability of cancer cells (notably SH-SY5Y neuroblastoma cells) while sparing healthy cells, and its combination with cisplatin produced synergistic cytotoxicity in neuroblastoma, breast, and prostate cancer cell lines. Mechanistically, at 20 µM, lercanidipine significantly inhibited MAPK signaling (~84% inhibition), induced apoptosis by activating caspase-3 and -8, and reduced the pro-inflammatory cytokine TNF-α. The 20 µM concentration was chosen for these mechanistic studies because higher doses (≥50 µM) caused excessive cytotoxicity; thus, 20 µM was identified as the optimal effective dose that allowed observation of molecular effects without non-specific cell death. Taken together, these results indicate that lercanidipine, beyond its primary cardiovascular uses, may be repurposed as a promising adjunct in cancer therapy—potentially improving treatment outcomes by directly promoting cancer cell death and attenuating tumor-promoting inflammation, especially when used in combination with standard chemotherapeutics such as cisplatin.

## Figures and Tables

**Table 1:** IC_50_ values of lercanidipine and positive control in HEK293, SH-SY5Y, PC3, and MCF7 cell lines.

Cpd No.	IC_50_ (μM)			
	HEK293	SH-SY5Y	PC3	MCF7
Lercanidipine	>150	31.48 ± 0.92	88.60 ± 1.39	107.54 ± 1.55
Cisplatin	2.98 ± 0.82	6.72 ± 0.89	5.78 ± 0.97	8.13 ± 0.95

**Table 2:** Cytotoxic effects of combined lercanidipine and cisplatin treatment.

Cell Lines	Fold Increase
SH-SY5Y	2.7
PC3	1.9
MCF7	1.6

**Table 3:** MAPK enzyme % inhibition values of lercanidipine and positive control (SB203580).

Cpd.	MAPK Inhibition (%)
Lercanidipine (20 µM)	83.60 ± 1.04
SB203580	99.80 ± 0.95

## Data Availability

All data used in this study are provided in the article and Supplementary Materials.

## Supplementary Materials

The following supporting information can be downloaded at https://www.mdpi.com/article/10.3390/ph18050651/s1.
